# A facile assay for zDHHC palmitoyl transferase activation elucidates effects of mutation and modification

**DOI:** 10.1016/j.jlr.2025.100743

**Published:** 2025-01-10

**Authors:** Naoko Adachi, Douglas T. Hess, Takehiko Ueyama

**Affiliations:** 1 Laboratory of Molecular Pharmacology, Biosignal Research Center, Kobe University, Kobe, Japan; 2 Department of Medicine, Case Western Reserve University School of Medicine, Cleveland, OH, USA

**Keywords:** cell signaling, enzymology-enzyme regulation, palmitoylation, acyltransferase, cancer mutations, fatty acyl-CoA, lipid signaling, tumor cell biology, zinc finger

## Abstract

At least 10% of proteins constituting the human proteome are subject to *S*-acylation by a long-chain fatty acid, thioesterified to a Cys thiol side chain. Fatty *S*-acylation (prototypically, *S*-palmitoylation) operates across eukaryotic phylogeny and cell type. *S*-palmitoylation is carried out in mammalian cells by a family of 23–24 dedicated zDHHC palmitoyl transferase enzymes, and mutation of zDHHCs is associated with a number of human pathophysiologies. Activation of the zDHHCs by auto-*S*-palmitoylation, the transthioesterification of the active site Cys by fatty acyl coenzyme A, is the necessary first step in zDHHC-mediated protein *S*-palmitoylation. Most prior in vitro assessments of zDHHC activation have utilized purified zDHHCs, a time- and effort-intensive approach, which removes zDHHCs from their native membrane environment. We describe here a facile assay for zDHHC activation in native membranes. We overexpressed hemagglutinin-tagged wild-type or mutant zDHHCs in cultured HEK293 cells and prepared a whole membrane fraction, which was incubated with fluorescent palmitoyl CoA (NBD-palmitoyl-CoA) followed by SDS-PAGE, fluorescence imaging, and Western blotting for hemagglutinin. We show by mutational analysis that, as assayed, zDHHC auto-*S*-palmitoylation by NBD-palmitoyl-CoA is limited to the active site Cys. Application of the assay revealed differential effects on zDHHC activation of posttranslational zDHHC modification and of zDHHC mutations associated with human disease, in particular cancer. Our assay provides a facile means of assessing zDHHC activation, and thus of differentiating the effects of zDHHC mutation and posttranslational modification on zDHHC activation versus secondary effects on zDHHC functionality including altered zDHHC interaction with substrate palmitoyl-proteins.

## Graphical Abstract

**Figure:**
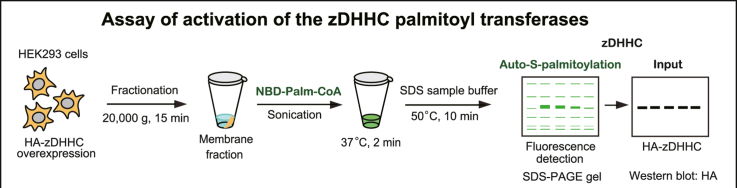

Eukaryotic proteins are subject to cotranslational and posttranslational covalent lipid modifications of disparate forms. Among these, long-chain fatty acid acylation, the thioesterification of a fatty acid to a Cys thiol sidechain (referred to by convention as *S*-palmitoylation, reflecting the most common form), is unique in that it is governed by dedicated enzymes that mediate in concert fatty acid addition (protein *S*-acyl transferases) and removal (acyl-protein thioesterases). *S*-palmitoylation is thereby dynamic, enabling a role in cellular signal transduction. *S*-palmitoylation has a broad functional purview including subcellular protein localization, protein-protein interactions, protein stability, intracellular protein trafficking, and cellular signal transduction (1, 2, 3, 4). Accordingly, *S*-palmitoylation subserves myriad aspects of cellular physiology, and dysregulated *S*-palmitoylation is associated with a broad spectrum of human pathophysiologies including neurological disorders and multiple forms of cancer (1, 5, 6).

The discovery in yeast of the zDHHC *S*-acyl transferases (7) revealed the enzymatic basis of protein *S*-palmitoylation, subsequently shown to operate across eukaryotic phylogeny and cell type. In mammals, 23–24 zDHHC enzymes have been identified, distinguished by subcellular localization and palmitoyl-protein substrate specificity (8, 9). More recently, it has been reported that in addition to the canonical, 16-carbon saturated fatty acid palmitate (16:0), at least some DHHCs may be capable of employing shorter- or longer-chain saturated or unsaturated fatty acids (14:0, 18:0, 18:1, 20.0) (10, 11).

Most zDHHCs are four-pass transmembrane proteins that contain two CCHC zinc fingers and the DHHC catalytic site in close proximity within the cytoplasmic loop. The zDHHCs are activated by auto-*S*-palmitoylation, the transthioesterification by fatty acyl coenzyme A (acyl-CoA) of the active site Cys, from which the fatty acyl moiety is transferred by the enzymatic transthioesterification to a target palmitoyl-protein Cys. zDHHC auto-*S*-palmitoylation is facilitated by a conserved hydrophobic cavity that positions the fatty acyl moiety of fatty acyl-CoA vicinal to the thiol sidechain of the zDHHC active site Cys (12, 13). The configuration of the binding pocket in various zDHHCs influences preference for fatty acyl-CoA chain length (10, 13, 14).

Most or all zDHHCs undergo posttranslational modification including phosphorylation as well as *S*-palmitoylation of Cys within the N-terminal and/or C-terminal cytoplasmic tails mediated by cognate zDHHC(s) (15, 16). Different point mutations within individual zDHHCs are associated with human disease including particular cancer. Assessment of the effects of posttranslational modification and mutation on zDHHC catalytic capacity requires assay of zDHHC auto-*S*-palmitoylation. Several approaches have been reported but in general are time- and effort-intensive and do not address zDHHCs in their native membrane environment (7, 14, 17, 18). NBD-palmitoyl-CoA, a palmitoyl-CoA derivative in which the palmitate moiety bears a fluorescent reporter N-[(7-nitro-2-1,3-benzoxadiazol-4-yl)-methyl]amino palmitoyl, has been used to detect auto-*S*-palmitoylation of purified zDHHCs in vitro (12, 17, 19). In this study, we developed a facile auto-S-palmitoylation assay for zDHHC activation using NBD-palmitoyl-CoA in native membranes. Hemagglutinin (HA)-tagged wild-type or mutant zDHHCs were overexpressed in cultured human HEK293 cells, from which a membrane fraction is prepared. Membranes are incubated with NBD-palmitoyl-CoA, then separated by SDS-PAGE followed by fluorescence imaging and Western blotting of HA for normalization with respect to expression levels. Here, we demonstrate this assay and show that it elucidates the effects of zDHHC posttranslational modification and disease-related point mutations on zDHHC activation.

## Materials and Methods

### Reagents and plasmids

All reagents were from Nacalai Tesque (Kyoto, Japan) unless otherwise specified. NBD-palmitoyl-CoA (16-NBD-16:0 Coenzyme A; 810705) was from Avanti Polar Lipids (Alabaster, AL), and 2BP (2-bromopalmitate; 238422) was from Merck (Darmstadt, Germany). pEF-Bos-HA-mouse zDHHC plasmids and pCEpuro-His6-Myc-human zDHHC plasmids were kindly provided by Dr Masaki Fukata (National Institutes of Natural Sciences, Aichi, Japan) and Dr Akio Kihara (Hokkaido University, Hokkaido, Japan), respectively. Mutations were individually introduced into zDHHC plasmids by site-directed mutagenesis [QuikChange Lightning Site-Directed Mutagenesis Kits, Agilent (Santa Clara, CA)]. All resulting plasmids were verified by DNA sequencing. HA was detected by Western blotting with HRP-tagged anti-HA mAb (Medical & Biological Laboratories, Tokyo, Japan: M180-7; RRID:AB_11124961), 1:2,500 for Western blotting. Myc was detected by Western blotting with anti-Myc mAb (Medical & Biological Laboratories, Tokyo, Japan: M047-3; RRID:AB_591112), 1:2000 for Western blotting. Horseradish peroxidase-conjugated secondary antibodies used to detect Myc labeling (RRID: AB_2307347) were from Jackson ImmunoResearch (West Grove, PA).

### Mammalian cell culture and transfection

HEK293 cells were maintained in minimum essential media with 10% fetal bovine serum, 100 units/ml penicillin, and 100 μg/ml streptomycin at 37°C in a humidified 5% CO_2_ atmosphere (2). Cells were transfected at ∼70% confluence using Lipofectamine 3,000 (Thermo-Fisher Scientific, Waltham, MA) according to the manufacturer's instructions.

### The in vitro auto-*S*-palmitoylation assay

HEK293 cells were cultured in 12-well plates and transfected with HA-tagged mouse zDHHCs or His_6_-Myc-tagged human zDHHCs and incubated for 24 h before harvesting. The cells were collected, washed in cold PBS, and sonicated in HN buffer (50 mM Hepes, 150 mM NaCl, pH 7.4) containing a protease inhibitor cocktail. For enrichment of membranes, the lysates were centrifuged at 20,000 *g* for 15 min. The resulting pellet was washed once with HN buffer and then resuspended by sonication in HN buffer containing NBD-palmitoyl-CoA. The samples were mixed at 37°C with 900 rpm agitation using a ThermoMixer (Eppendorf, Hamburg, Germany) for the indicated time points. The reaction was quenched with SDS sample buffer at 50°C for 10 min, followed by SDS-PAGE and fluorescence laser scanning and Western blotting. For zDHHCs with low NBD-palmitate binding affinity, HEK293 cells overexpressing tagged zDHHCs were incubated in minimum essential medium containing 1% fatty acid–free BSA for 1 h prior to harvesting. Membrane fractions were then separated by centrifugation, beginning with a clarification spin (900 *g* for 3 min) to remove nuclei and cell debris, followed by centrifugation at 20,000 *g* for 15 min. These starvation conditions and the clarification spin enhanced signal-to- noise of auto-*S*-palmitoylation signals, whereas these additional steps had little impact when assaying more robustly labeled zDHHCs including zDHHC5 (supplemental Fig. S1B).

### Data acquisition and presentation

Western blotting signals were detected using the FUSION SOLO S system, and densitometric analysis was performed with Evolution Capt software (Vilber, Paris, France). NBD-palmitate fluorescence was detected and quantified using a Typhoon FLA 9500 fluorescent laser scanner (GE HealthCare Life Sciences, Chicago, IL) or a FUSION SOLO S system (Vilber, Paris, France). All fluorescence signals were normalized with respect to zDHHC expression as assessed by Western blotting.

All quantified data are presented as mean ± S.D. Comparisons between groups using Student’s *t* test or one-way ANOVA with Dunnett’s or Tukey’s posthoc test as appropriate were conducted using GraphPad Prism 7 (GraphPad, La Jolla, CA). *P* < 0.05 was considered significant.

## Results

### Auto-*S*-palmitoylation assay of zDHHC activation with zDHHC5 as prototype

zDHHC auto-*S*-palmitoylation directly reflects zDHHC *S*-acyl transferase catalytic capacity. We validated and parameterized our auto-*S*-palmitoylation assay and assessed its utility in elucidating the effects of zDHHC posttranslational modification using zDHHC5 as prototype. zDHHC5 is localized largely to plasma and endosomal membranes, where it participates in multiple aspects of cellular function in multiple cell types (20, 21, 22, 23).

**Fig. 1:**
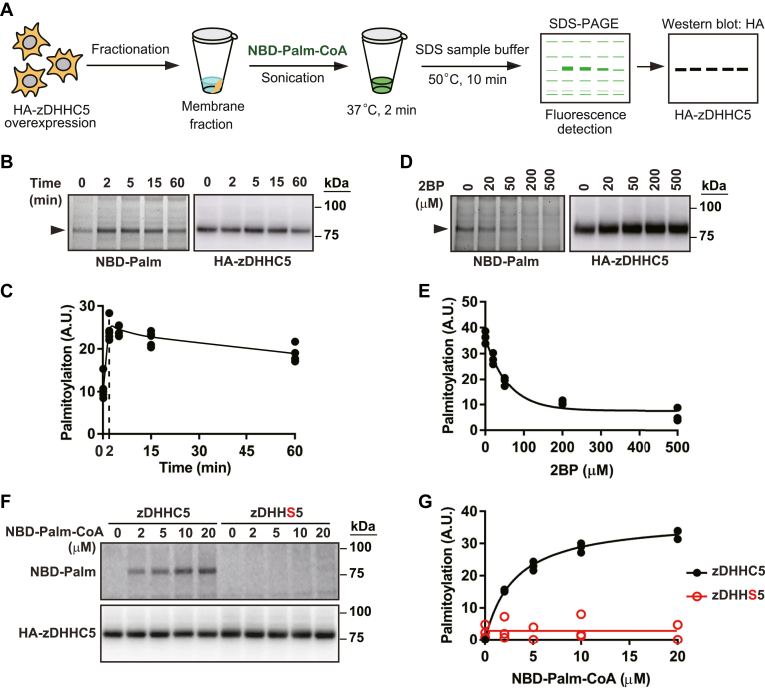
Validation of the auto-*S*-palmitoylation assay with zDHHC5 as exemplar. A: A schematic illustration of the standard assay protocol. B and C: NBD-palmitate (10 μM) labeling as assessed by fluorescence imaging was maximal after 2 min incubation with NBD-palmitoyl-CoA and largely maintained over 60 min. Western blotting for HA demonstrated relative levels of zDHHC5 expression, used for normalization of fluorescence signals. Note that fluorescence imaging and Western blotting were carried on the same gel. D and E: Incubation of membrane samples for 10 min with 2-bromopalmitate (2BP) prior to addition of NBD-palmitoyl-CoA in vitro eliminated NBD-palmitate labeling in a concentration-dependent fashion (see also supplemental Fig. S1B, C). F and G: Mutation of the active site Cys to Ser eliminated auto-*S*-palmitoylation.

As depicted schematically in Fig. 1A, HA-tagged mouse zDHHC5 was overexpressed in cultured HEK293 cells by transfection with pEF-Bos-HA-mouse zDHHC5 plasmid. Cells were collected, and a total membrane fraction was generated by probe sonication in isotonic buffer followed by centrifugation at 20,000 *g*. The resultant pellets, containing 15–20 μg total protein, were dispersed by probe sonication following the addition of 50 μl isotonic buffer containing NBD-palmitoyl-CoA. Samples were incubated with continuous agitation until quenched with SDS-PAGE loading buffer then run on SDS-PAGE gels followed by detection of zDHHC5-bound NBD-palmitate by fluorescence imaging and by Western blotting for HA.

We first examined the effects of NBD-palmitoyl-CoA concentration and incubation time. As shown in supplemental Fig. S1A, incubation (2 min) of transfected or untransfected samples with increasing concentrations of NBD-palmitoyl-CoA resulted in the appearance of multiple NBD-labeled species at higher concentrations of NBD-palmitoyl-CoA, but at all concentrations (2–100 μM) in samples from zDHHC5-transfected cells, zDHHC5 was easily identified in fluorescent images as a prominent band of 78 kDa, as confirmed by Western blotting for HA. The signal-to-noise ratio was not improved by adding a clarification spin (900 *g* for 3 min) to remove nuclei and cell debris prior to centrifugation at 20,000 *g* for 15 min (supplemental Fig. S1B). As shown in Fig. 1B, C, labeling increased rapidly over the first 2 min of incubation and declined only slightly over the next hour. We subsequently employed 10 μM NBD-palmitoyl-CoA with 2 min incubation as our standard condition.

We verified that palmitoyl-NBD labeling of zDHHC5 identified auto-*S*-palmitoylation with the covalent *S*-palmitoylation inhibitor 2BP, known to bind at the active site of zDHHCs (12). As shown in Fig. 1D, E, preincubation (10 min) of membrane samples with 2BP in vitro*,* prior to addition of NBD-palmitoyl-CoA, effectively suppressed NBD labeling. In addition, as shown in supplemental Fig. S1C, D, incubation of intact cells with 2BP (100 μM, 2 h) prior to preparation of the membrane fraction effectively eliminated subsequent NBD labeling.

Assay of zDHHC auto-*S*-palmitoylation that accurately assesses zDHHC *S*-acyl transferase catalytic capacity requires that the assay reports active site and not other *S*-palmitoylation, and zDHHC5 contains Cys other than the active site Cys that could in principle be subject to auto-*S*-palmitoylation, which might not be differentiated by 2BP. Therefore, we carried out a mutational analysis in which the active site Cys was converted to Ser. As shown in Fig. 1F, active site Cys mutation (DHHS: Cys134Ser) eliminated palmitoyl-NBD labeling of zDHHC5 under the assay conditions employed. Thus, our assay specifically reveals active site auto-*S*-palmitoylation and therefore zDHHC5 catalytic capacity.

Finally, in order to facilitate comparison between assays of various zDHHCs and between the effects of various mutations of a given zDHHC, we calculated Michaelis-Menten kinetic constants following incubation with increasing concentrations of NBD-palmitoyl-CoA. For wild-type zDHHC5 (see Fig. 1G), the *K*_*m*_ was 3.59 ± 0.29 μM, and the *V*_*max*_ was 39.55 ± 1.00 (n = 12).

### Auto-*S*-palmitoylation assay of zDHHC5 with function-altering mutations

**Fig. 2:**
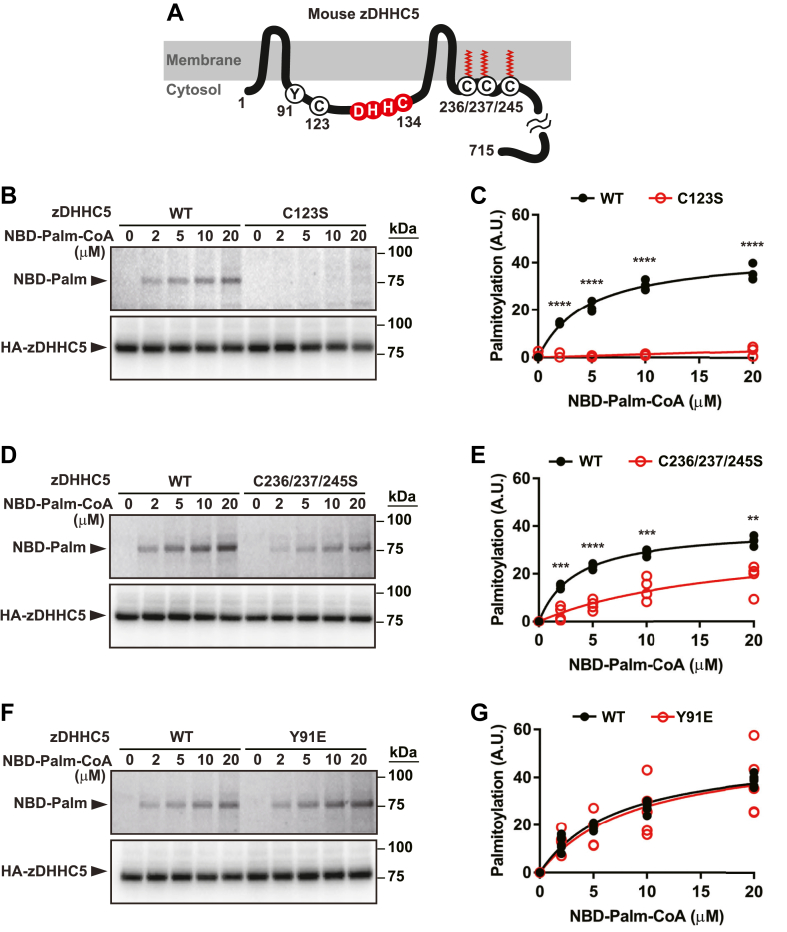
Analysis of the effects of potential function-altering mutations of zDHHC5 on auto-*S*-palmitoylation. A: A schematic illustration of the topology of zDHHC5. Highlighted are Tyr91, a reported site of phosphorylation; Cys123, which participates in the second zinc-finger domain; the active site DHHC motif; the cluster of three Cys within the C-terminal cytoplasmic tail that are reported to undergo enzymatic *S*-palmitoylation. B and C: C123S mutation eliminates auto-*S*-palmitoylation (n = 3). D and E: Cys→Ser mutation of C236/237/245, which would abrogate enzymatic *S*-palmitoylation within the C-terminal cytoplasmic domain, significantly diminishes auto-*S*-palmitoylation (n = 4). F and G: Phosphomimetic Tyr91→Glu91 mutation had no effect on auto-*S*-palmitoylation (n = 6). All assays were conducted with 2 min incubation with NBD-palmitoyl-CoA. In (C, E, G), ∗∗, ∗∗∗, and ∗∗∗∗ represent respectively *P* < 0.01, *P* < 0.001, and *P* < 0.0001 by Student’s *t* test.

As for most zDHHCs, zDHHC5 is a 4-pass transmembrane protein with two zinc finger domains and the DHHC active site located within the single cytoplasmic loop (Fig. 2A). It is well established that disruption of the zinc finger motifs leads to a loss of function of zDHHCs, resulting from alteration of the configuration of the DHHC active site that prevents auto-*S*-palmitoylation (19, 24). Application of the auto-*S*-palmitoylation assay revealed that, notably, mutation (Cys-Ser) of a single Cys, Cys123 within the second zinc finger domain (C120-C123-H133-C140) abrogated auto-*S*-palmitoylation of zDHHC5 in its native membrane environment (Fig. 2B, C), pointing to the potential utility of the auto-*S*-palmitoylation assay as a component of zDHHC structure-function analysis.

It has been shown that zDHHC5 is posttranslationally modified by *S*-palmitoylation of a cluster of Cys within the C-terminal cytoplasmic tail (Cys236/237/245) (15), and it has been reported that *S*-palmitoylation enhances the binding of zDHHC5 to the substrate palmitoyl-protein phospholemman (23) as well as to the function-enhancing accessory protein Golga7b (22). We employed the auto-*S*-palmitoylation assay to examine the effect of C-terminal cytoplasmic tail *S*-palmitoylation on zDHHC5 catalytic capacity.

As shown in supplemental Fig. S2A, B, we verified that zDHHC5 overexpressed in HEK293 cells undergoes *S*-palmitoylation within its C-terminal cytoplasmic tail by directly assessing the levels of *S*-palmitoylation in wild-type zDHHC5 and zDHHC5 with Cys to Ser mutations at Cys236/237/245, using the acyl-RAC assay (25) and metabolic labeling combined with click chemistry (26, 27). We confirmed that Cys236/237/245 are the primary *S*-palmitoylation sites outside the active site, as the combination of active site and Cys236/237/245Ser mutations reduced *S*-palmitoylation levels to less than 10% of the wild-type zDHHC5. As shown in Fig. 2D, E, application of the auto-*S*-palmitoylation assay revealed that the lack of *S*-palmitoylation in the C-terminal cytoplasmic tail suppressed auto-*S*-palmitoylation (*K*_*m*_ 17.95 ± 11.12 μM, *V*_*max*_ 35.25 ± 12.51). It should be noted that cellular levels of free fatty acyl-CoA are likely to be significantly lower than 1 μM (28) and that suppression of auto-*S*-palmitoylation in the absence of C-terminal cytoplasmic tail *S*-palmitoylation in the native membrane environment was virtually complete at lower, more physiological concentrations of NBD-palmitoyl-CoA. This finding, which is consistent with previous results with zDHHC9 purified from its native membrane environment (19), points to a positive, allosteric influence of C-terminal cytoplasmic tail *S*-palmitoylation on auto-*S*-palmitoylation that may reflect alteration in the configuration of the fatty acyl-CoA biding pocket, which has been shown by structural analyses to depend upon the precise topology of the four transmembrane segments flanking the cytoplasmic loop (12, 13).

Tyrosine phosphorylation of Y91 within the cytoplasmic loop by the Src kinase LYN has been associated with suppressed *S*-palmitoylation by zDHHC5 of downstream target palmitoyl-proteins (21). Suppression of zDHHC5 activity was observed with the phospho-mimetic Y91E mutant. However, as shown in Fig. 2F, G, we could detect no change in auto-*S*-palmitoylation of Y91E versus wild-type zDHHC5. This finding suggests that phosphorylation of Tyr91 may suppress zDHHC5-mediated protein *S*-palmitoylation in significant part by altering the interaction of activated zDHHC5 with palmitoyl-protein substrates.

### Application of the auto-*S*-palmitoylation assay across the family of zDHHCs

**Fig. 3:**
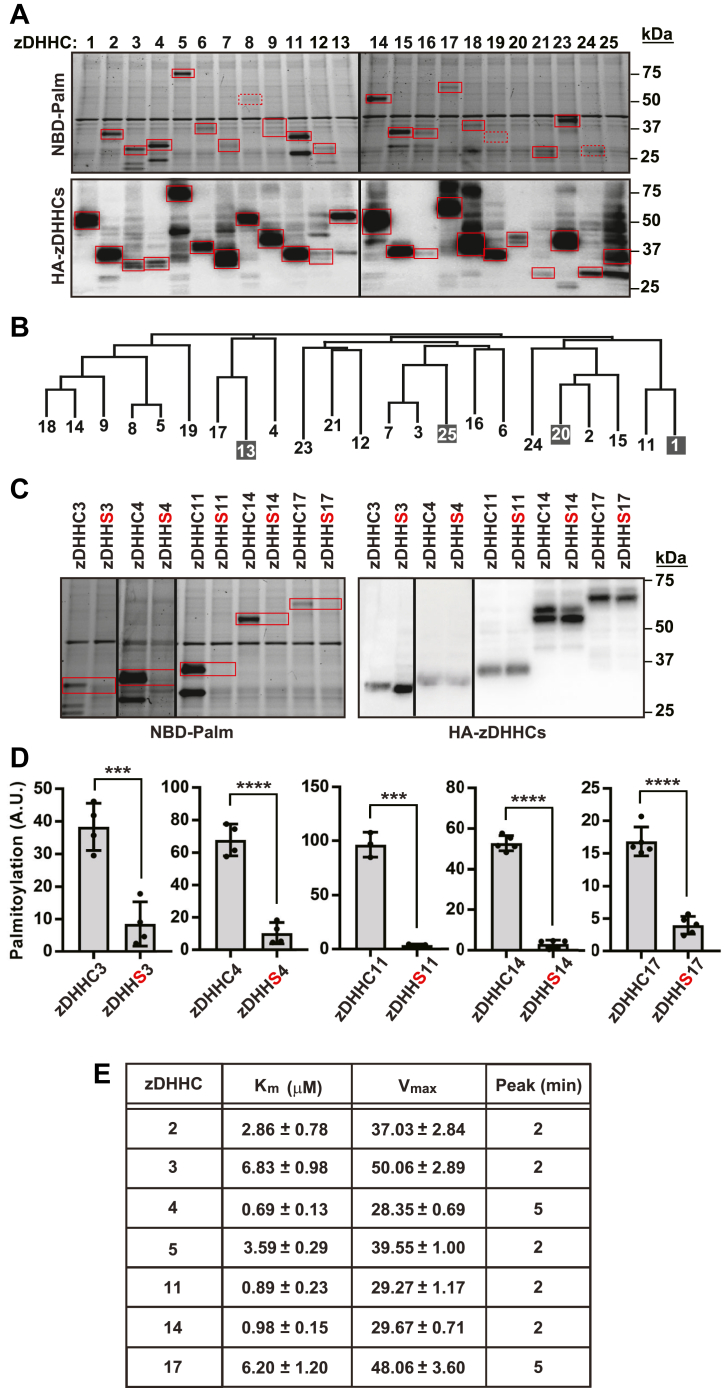
Application of the auto-*S*-palmitoylation assay across the family of zDHHCs. A: Raw data (from 1 of 4 replications) illustrating NBD-palmitate binding to the 23 mouse zDHHCs and corresponding HA Western blotting. Solid black vertical lines delineate two identical gels run in parallel. B: A phylogenetic tree of the zDHHCs based on the complete sequence of the intracellular loop containing the DHHC active site. Dark boxes highlight the four DHHCs that expressed as assessed by HA blotting but were not labeled by NBD-palmitoyl-CoA. C and D: Cys→Ser mutation of the active site Cys in a representative set of zDHHCs (zDHHC3/4/11/14/17) eliminates labeling by NBD-palmitoyl-CoA. In D, error bars represent S.D. (n = 3–5); ∗∗∗ and ∗∗∗∗ indicate respectively *P* < 0.001 and *P* < 0.0001. All assays were conducted with 5 min incubation with 10 μM NBD-palmitoyl-CoA. E: A table shows Michaelis-Menten values (see supplemental Fig. S5 for original data) and time to maximal (peak) labeling (see supplemental Fig. S4 for original data) for a representative set of seven zDHHCs (distributed across subgroups; see Fig. 3B).

We individually expressed each of the 23 mouse zDHHCs and assessed auto-*S*-palmitoylation under standard conditions (10 μM NBD-palmitoyl-CoA, 2 min incubation). Under these conditions, labeling was detected in 16 out of 23 zDHHCs, as shown in Fig. 3A, which presents representative raw data. To improve signal detection, we combined depletion of endogenous acyl-CoA with higher concentrations of NBD-palmitoyl-CoA (25 μM) and performed a clarification spin to remove nuclei and cell debris, thereby reducing background noise. These additional steps enabled the detection of fluorescence signals from zDHHC8, zDHHC19, and zDHHC24, while also enhancing the relatively weak signals observed under standard conditions (supplemental Fig. S3). Using zDHHC3, zDHHC4, zDHHC11, zDHHC14, and zDHHC17, which distribute across the zDHHC family, we confirmed through C→S mutation that, as observed for zDHHC5 (Fig. 1F), labeling was confined to the active site Cys, presumably due to high reactivity of the active site thiol and/or the precise juxtaposition of target thiol and acyl donor provided by the hydrophobic acyl-CoA—binding channel that targets only the active site for modification (Fig. 3C, D).

Although zDHHC1, zDHHC13, zDHHC20, and zDHHC25 were expressed as assessed by HA Western blotting, they were not labeled by NBD-palmitoyl-CoA (Fig. 3A). Thus, 19/23 zDHHCs were susceptible to assay. As shown in the phylogenetic tree presented in Fig. 3B [Clustal Omega], the zDHHCs recalcitrant to assay are distributed across multiple subgroups. The mass of NBD-palmitoyl-CoA (∼1,234 Da) is ∼17% greater than the mass of palmitoyl-CoA (∼1,056 Da), and NBD is a bulky group in comparison to straight-chain fatty acid. Thus, the absence of labeling of zDHHC1, zDHHC13, zDHHC20, and zDHHC25 may point to a more restrictive fatty acyl-CoA binding pocket in those species. Whether this might reflect different endogenous fatty acyl-CoA substrate preferences remains to be determined. Generally, although our assay is applicable for the majority of zDHHCs, alternative labels may optimize coverage of the zDHHCs.

We examined the time-course of labeling with NBD-palmitoyl-CoA (10 μM) in a representative set of zDHHCs (zDHHC2, 3, 4, 11, 14, and 17). As shown in supplemental Fig. S4, labeling was maximal within 2 min in most cases (Fig. 3E) as for DHHC5 (Fig. 1C). For zDHHC4 and DHHC17, peak labeling was observed at 5 min (Fig. 3E). The stability of labeling varied substantially. Labeling of zDHHC14 and zDHHC17 was largely preserved over the 60 min time course examined (supplemental Fig. S4J, L), as for zDHHC5 (Fig. 1C), whereas labeling of zDHHC2, 3, 4, and 11 declined substantially after 5 min. The decline in labeling may reflect transfer of NBD-palmitate to substrate palmitoyl proteins and/or hydrolysis of the active site thioester linkage (14, 18).

Finally, we examined labeling with increasing concentrations of NBD-palmitoyl-CoA of a representative set of zDHHCs: zDHHC 2, 3, 4, 11, 14, and 17, to compare labeling kinetics. As shown in supplemental Fig. S5 and summarized in Fig. 3E, *K*_*m*_ values varied over an ∼10-fold range, whereas *V*_*max*_ values were substantially less variable (∼1.8-fold range). These results point to differences in the affinity of individual zDHHCs for NBD-palmitoyl-CoA that may be relevant in optimizing assay conditions for a given zDHHC.

### Auto-*S*-palmitoylation assay of zDHHCs bearing cancer-associated point mutations

Altered expression of zDHHCs has been associated with tumorigenesis and tumor progression in multiple cancers, consistent with the reported role of *S*-palmitoylation in regulating the function of multiple oncogene products and tumor suppressors (5, 6). A role for altered zDHHC function in cancer is also suggested by the association in multiple cancers of point mutations of various zDHHCs (1). However, the functional impact of those mutations remains largely unexplored, and we applied the auto-*S*-palmitoylation assay to determine their effect on zDHHC catalytic capacity. These included seven mutations in four zDHHCs: S306F mutation in zDHHC2 [hepatocellular carcinoma (29)], M356I mutation in zDHHC2 and N44D mutation in zDHHC7 [colorectal cancer (29, 30)], P104S mutation in zDHHC4 [breast cancer (30)], and three mutations in zDHHC14: C181Y and A373Y [prostate cancer (31)] as well as L215S [testicular germ cell tumor (31)].

We overexpressed wild-type and mutant human Myc-tagged zDHHCs in HEK293 cells employing the pCEpuro-His6-Myc-1 vector. Sample processing and the auto-*S*-palmitoylation assay were carried out as for mouse zDHHCs.

**Fig. 4:**
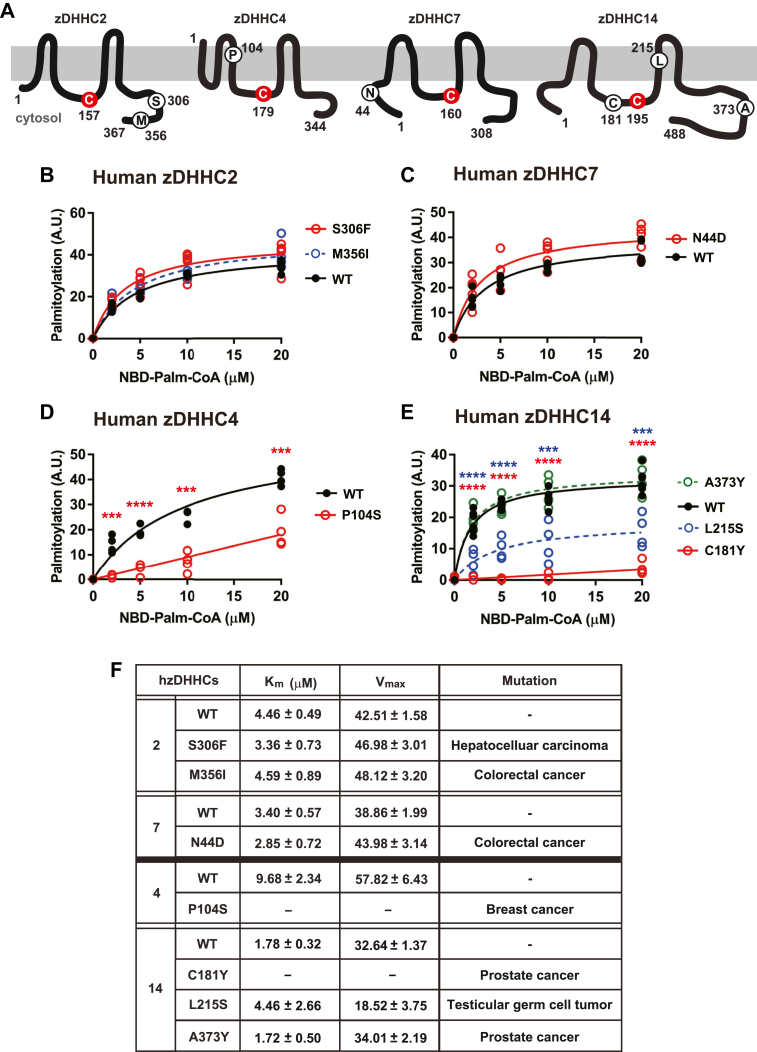
Auto-*S*-palmitoylation assay of zDHHCs bearing cancer-associated point mutations. A: A schematic illustration of the topologies of four human zDHHCs in which point mutations have been associated with cancer (zDHHC2/4/7/14). For each zDHHC, the catalytic Cys is indicated in red, and residues endogenously mutated in association with various cancers are circled in black. As shown in (B–E), these mutations were introduced in human zDHHCs, and their effect on auto-*S*-palmitoylation was assessed. Original data are shown in supplemental Fig. S6. B: Neither S306 F nor M356I mutation within the C-terminal cytoplasmic tail of zDHHC2 affected auto-*S*-palmitoylation (n = 5). C: N44D mutation within the N-terminal cytoplasmic tail of zDHHC7 had no effect on auto-*S*-palmitoylation (n = 4). D: P104S mutation within the third transmembrane segment of zDHHC4 significantly diminished auto-*S*-palmitoylation. E: A373Y mutation within the C-terminal cytoplasmic tail had no effect on auto-*S*-palmitoylation, whereas C181Y mutation within the second zinc finger eliminated auto-*S*-palmitoylation. L215S mutation within the third transmembrane segment substantially diminished auto-*S*-palmitoylation. (n = 4–6). zDHHC2, zDHHC7, and zDHHC14 were incubated with NBD-palmitoyl-CoA for 2 min, while zDHHC4 was incubated with NBD-palmitoyl-CoA for 5 min. F: A table displays the results of Michaelis-Menten analysis of the data shown in (B–E) and identifies the cancers in which zDHHC point mutations have been reported. In 4 B and 4E, data were assessed by one-way ANOVA with posthoc Tukey’s test. In (C and D), data were assessed by Student’s *t* test. In (D and E), ∗∗∗ and ∗∗∗∗ indicate *P* < 0.001 and *P* < 0.0001, respectively.

As shown in Fig. 4B, F and in supplemental Fig. S6A where representative raw data are shown, neither S306 F nor M356I mutation of zDHHC2 within the C-terminal cytoplasmic tail (Fig. 4A) had an effect on auto-*S*-palmitoylation. Similarly, N44D mutation within the N-terminal cytoplasmic tail of zDHHC7 (Fig. 4A) had no effect (Fig. 4C, F and supplemental Fig. S6B).

In contrast, P104S mutation within the third transmembrane segment of zDHHC4 (Fig. 4A) resulted in greatly diminished auto-*S*-palmitoylation, which was essentially eliminated at lower NBD-palmitate concentrations (2–10 μM) (Fig. 4D, F and supplemental Fig. S6C). A373Y mutation within the N-terminal cytoplasmic tail of zDHHC14 (Fig. 4A) had no effect (Fig. 4E, F and supplemental Fig. S6D), whereas C→Y mutation of Cys181 within the second zinc finger domain (Fig. 4A) eliminated auto-*S*-palmitoylation (Fig. 4E, F and supplemental Fig. S6D), consistent with abrogation of auto-*S*-palmitoylation of zDHHC5 by mutation of a single Cys within the second zinc-finger domain (Fig. 2B, C). L215S mutation within the third transmembrane segment of zDHHC14 (Fig. 4A) substantially diminished auto-*S*-palmitoylation (∼60% increase in K_m_ and ∼43% decrease in V_max_) (Fig. 4E, F and supplemental Fig. S6D).

Thus, the auto-*S*-palmitoylation assay discriminates between cancer-related zDHHC mutations that do or do not impact zDHHC activation and provides novel insight into the potential role of diminished zDHHC activation in breast cancer (zDHHC4), as well as prostate cancer and testicular germ cell tumor (zDHHC14).

## Discussion

Activation of the zDHHC palmitoyl-transferases by auto-*S*-palmitoylation is the essential first step in enzymatic protein *S*-palmitoylation, a primary posttranslational protein modification in all eukaryotes. We present here a facile assay for zDHHC auto-*S*-palmitoylation that assesses activation in the native membrane environment, without enzyme purification that compromises physiological relevance. We show that, under the conditions employed, the assay is specific for active site Cys and is applicable for the majority of zDHHCs. Application of the assay provides insight into the role of zDHHC posttranslational modification and in particular of enzymatic *S*-palmitoylation in regulation of zDHHC catalytic capacity and elucidates the effects on zDHHC activation of cancer-related point mutations.

Multiple zDHHCs have been shown to be subject to enzymatic *S*-palmitoylation by cognate zDHHCs at sites separate from the catalytic Cys, within the N-terminal and/or C-terminal cytoplasmic domains (15, 32). *S*-palmitoylation within the C-terminal cytoplasmic domain has been reported for zDHHC5/6/8/9/16/17/20 (15, 19, 32). The effect of enzymatic *S*-palmitoylation on zDHHC activation has not been examined in most cases. Our results with zDHHC5 (Fig. 2D, E), which are consistent with prior analysis of zDHHC9 purified from its native membrane environment (19), indicate that *S*-palmitoylation within the C-terminal cytoplasmic domain serves as a positive allosteric modulator of auto-*S*-palmitoylation and suggests the likelihood that the activity of zDHHCs subject to enzymatic *S*-palmitoylation is regulated by dynamic cycles of *S*-palmitoylation/depalmitoylation (23, 33, 34).

Our assay has certain limitations. We assessed auto-*S*-palmitoylation in HEK293 cells overexpressing zDHHCs. Significant expression of most transfected zDHHCs was not seen in other cell lines we examined, which typically exhibit lower transfected protein expression levels than HEK293 cells. We also found that cells overexpressing zDHHCs must be freshly harvested for assay: storage at −80°C significantly reduces NBD-palmitoyl-CoA affinity. Effectively low levels of zDHHC expression in native cells, and restrictions on storage conditions, preclude application of our assay to native tissues including clinical samples. In addition, although our mutational analysis indicated that only the active site DHHC Cys was subject to auto-*S*-palmitoylation by NBD-palmitoyl-CoA, active-site Cys mutants should be assessed as an essential control when applying our assay. Furthermore, potential differences in kinetic behavior between NBD-palmitoyl-CoA and endogenous palmitoyl-CoA must be considered, as NBD is a bulky group compared to a straight-chain fatty acid.

Dysregulated, generally diminished, protein *S*-palmitoylation is associated with a broad spectrum of human diseases. In particular, dysregulated *S*-palmitoylation of multiple oncogenes and tumor suppressors in multiple forms of cancer has been associated with pathophysiological alterations in cancer cell proliferation and survival, cell invasion and metastasis, and antitumor immune responsivity (5, 6). *ZDHHC14* was identified as the product of a tumor suppressor gene, with decreased *ZDHHC14* levels in testicular germ cell tumors and prostate cancer tissue samples and cell lines (31). Consistent with this, we confirmed that *ZDHHC14* mRNA levels are downregulated in prostate cancer tissues compared to adjacent normal tissues in two independent GEO datasets (supplemental Fig. S7). In addition to the downregulation of *ZDHHC* expression, missense and deletion mutations of one or more*ZDHHC* genes including *ZDHHC14* has been associated with a number of varied cancers (1). However, the specific substrate related to tumorigenesis or the effects of mutation on zDHHC functionality remain largely unexplored. Here, we examined the effects on auto-*S*-palmitoylation of seven cancer-associated mutations in four zDHHCs.

We found that neither S306 F (hepatocellular carcinoma) nor M356I (colorectal cancer) mutation within the C-terminal cytoplasmic tail of zDHHC2 affected auto-*S*-palmitoylation. Similarly, N44D mutation (colorectal cancer) within the N-terminal cytoplasmic tail of zDHHC7 had no effect. In contrast, P104S mutation (breast cancer) within the third transmembrane segment of zDHHC4 substantially diminished auto-*S*-palmitoylation, as did L215S mutation (testicular germ cell tumor) in the third transmembrane segment of zDHHC14. Structural modeling (UniProt: https://www.uniprot.org/) shows that P104 in zDHHC4 and L215 in zDHHC14, both nonpolar and hydrophobic amino acids, are constituents of the transmembrane helices that form the hydrophobic fatty acyl-CoA–binding pocket. We hypothesize that mutation of these residues to serine, a polar and hydrophilic amino acid, would reduce the hydrophobicity of the binding pocket enough to diminish the binding affinity for fatty acyl-CoA, leading to decreased auto-*S*-palmitoylation. In zDHHC14, A373Y mutation (prostate cancer) within the C-terminal cytoplasmic tail had no effect. C181Y mutation within the second zinc finger eliminated auto-*S*-palmitoylation, as did mutation of a Cys within the second zinc finger of zDHHC5 shown here. Thus, our results provide new insight into the effects of zDHHC mutation in breast and prostate cancer and in testicular germ cell tumor.

One example is represented by our finding that a cancer-related mutation of zDHHC4 distant from the active site (Pro104) greatly suppressed zDHHC activation. zDHHC4 functions as a palmitoyltransferase for KAI1 (CD82), a metastasis suppressor, promoting its localization to lipid rafts on pericyte membranes. This enables KAI1 to inhibit VEGF and PDGF, suppressing angiogenesis. Palmitoylated KAI1 also activates the Src/p53 pathway, inducing leukemia inhibitory factor production, which further reduces angiogenesis by targeting endothelial cells and pericytes (35). Therefore, loss of zDHHC4 activity would promote pathological angiogenesis and accelerate breast cancer progression. We anticipate that further analysis of disease-related mutations in zDHHCs, aided by our assay, will expand understanding of the roles of dysregulated protein *S*-palmitoylation in pathophysiology.

Our findings illustrate the utility of the auto-*S*-palmitoylation assay in elucidating the functional effects of disease-related zDHHC mutation. Mutations of zDHHCs have been associated with a wide spectrum of neurological conditions, including schizophrenia, autism, Parkinson's disease, amyotrophic lateral sclerosis, mental retardation, and Raymond-type syndromic X-linked intellectual disability (1). Generally, however, the effect of mutation on zDHHC functionality remains unknown. Our assay of zDHHC auto-*S*-palmitoylation in native membrane provides a facile and physiologically relevant approach to assessing the effects of zDHHC mutation associated with neurological disease and with other pathophysiologies in which dysregulated protein *S*-palmitoylation may reflect altered zDHHC activity. In addition, our assay provides a facile means with which to assess the effects of posttranslational modification on the catalytic capacity of the zDHHCs in their native membrane environment.

## Data availability

All data are available in this manuscript.

## Supplemental data

This article contains supplemental data.

## Conflict of interest

The authors declare that they have no conflicts of interest with the contents of this article.
