# Supplementary material for: A facile assay for zDHHC palmitoyl transferase activation elucidates effects of mutation and modification
Source: J Lipid Res. 2025 Jan 10;66(2):100743. doi: 10.1016/j.jlr.2025.100743 (PMC11870023; doi:10.1016/j.jlr.2025.100743)
Supplement: Supplemental data [file mmc1.docx]

**Supplemental data**

**Supplemental Figure Legends**

**Figure S1.** ***Validation of the auto-S-palmitoylation assay of zDHHC5.*** *A*, Increasing concentrations of NBD-palmitoyl-CoA (2 min incubation) resulted in enhanced fluorescence signals from multiple proteins in both zDHHC5 transfected and untransfected cells. At all concentrations of NBD-palmitoyl-CoA, zDHHC5 is easily identified in fluorescence scans as a transfection-dependent band of 78 kDa, confirmed by Western blotting for HA. *B*, A clarification spin (900 *g* for 3 min) prior to centrifugation at 20,000 *g* for 15 min to remove nuclei and cell debris slightly reduces background signals. Total protein content is visualized using Coomassie Brilliant Blue (CBB) staining. *C and D*, Treatment of intact cells with 2BP in DMSO (100 µM, 2 h) before harvesting and assay greatly diminished auto-*S*-palmitoylation of zDHHC5. This assay was conducted with 2 min incubation with 10 µM NBD-palmitoyl-CoA. In S1C, data (n = 3) were analyzed by two-tailed Student’s *t*-test and ***indicates *P* < 0.001. Arrowheads indicate HA-zDHHC5.

**Figure S2**. ***Verification of S-palmitoylation within the C-terminal cytoplasmic tail of zDHHC5.*** Cell extracts were subjected to the acylRAC assay (A), and metabolic labeling by 17-octadecynoic acid (17ODYA) as detected by bio-orthogonal click chemistry (B) for *S*-palmitoylation, carried out as described in Supporting Experimental Procedures. *S*-palmitoylation was assessed in wild-type zDHHC5 and in zDHHC5 subjected to C236/237/245S mutation to remove the Cys within the C-tail cytoplasmic domain that are reportedly *S*-palmitoylated. In addition, we examined the effects of mutation of the active site Cys (DHHC→DHHS). C236/237/245S mutation significantly diminished *S*-palmitoylation. *S*-palmitoylation was also diminished substantially in the active site mutant (DHHS), presumably reflecting basal active site *S*-palmitoylation. Levels of *S*-palmitoylation in the combined C236/237/245S and DHHS mutants were less than 10% of wild-type. Data were analyzed by ANOVA with post-hoc Tukey’s test (**P* < 0.05, *****P* < 0.0001). Error bars represent standard deviation (S.D.); n = 3 in A, n = 4 in B.

***Figure S3. Application of the auto-S-palmitoylation assay to*** ***zDHHCs with low NBD-palmitate binding affinity.*** Representative raw data (from one of three replicates) illustrating NBD-palmitate binding to twelve mouse zDHHCs, along with corresponding HA Western blot analysis. HEK293 cells overexpressing tagged zDHHCs were incubated in minimum essential medium containing 1% fatty acid-free BSA for 1 h prior to harvesting. Membrane fractions were separated by centrifugation, beginning with a clarification spin (900 *g* for 3 min) to remove nuclei and cell debris, prior to centrifugation at 20,000 g for 15 min. The samples were incubated with 25 µM NBD-palmitoyl-CoA for 2 min, resulting in detectable fluorescence signals for zDHHC8, zDHHC19, and zDHHC24, as well as enhanced fluorescence signals for zDHHC7, zDHHC9, zDHHC12, zDHHC16, and zDHHC18. Auto-*S*-palmitoylation was not detected for zDHHC1, zDHHC13, zDHHC20, or zDHHC25 under these conditions.

**Figure S4. *Time-course of NBD-palmitate labeling*.** A representative set of six zDHHCs (HA-tagged) was evaluated (zDHHC2/3/4/11/14/17). Samples were incubated with 10 µM NBD-palmitoyl-CoA for the indicated times. NBD-palmitate labeling was detected by fluorescence imaging, and levels of zDHHC expression were evaluated by Western blot for HA. Labeling was maximal at 2 min, the shortest time-point examined, or 5 min (zDHHC4 and DHHC17). The stability of labeling varied greatly across the zDHHCs. zDHHC2, n = 4; zDHHC3, n = 3; zDHHC4, n = 4; zDHHC11, n = 3; zDHHC14, n = 4; zDHHC17, n = 4.

**Figure S5. *Kinetics of NBD-palmitate binding.*** Samples (zDHHC2/3/4/11/14/17 as in Fig. S4) were incubated for 2 min (or 5 min in the case of zDHHC4/17) with NBD-palmitoyl-CoA over a concentration range of 0-20 µM. In A-L, raw data are shown above, and binding curves are shown below. Figure S5B, n = 4; for all other zDHHCs, n = 3. Michaelis-Menten values are provided in Figure 3E.

**Figure S6. *Application of the auto-S-palmitoylation assay to zDHHCs with cancer-related point mutation(s).***  The effects of seven mutations in four zDHHCs were assessed. Quantified data are presented in Figure 4. *A*, In zDHHC2, neither S360F nor M356I mutation affected auto-*S*-palmitoylation. *B*, N44D mutation in zDHHC7 also had no effect. *C*, P104S mutation in zDHHC4 greatly diminished auto-S-palmitoylation. *D*, In zDHHC14, A373Y mutation had no effect, whereas C181Y mutation eliminated auto-*S*-palmitoylation. L251S mutation substantially diminished auto-*S*-palmitoylation. Solid black vertical lines delineate two identical gels run in parallel.

**Figure S7. mRNA expression of *ZDHHC14* is reduced in prostate cancer tissues.** Human data taken from GEO datasets (GEO: GSE133626 (*A-C*) and GSE104131 (*D-F*)), showing significant downregulation of *ZDHHC14* mRNA expression in prostate cancer tissues compared with adjacent normal tissues. Glyceraldehyde 3-phosphate dehydrogenase (*GAPDH*) and hypoxanthine guanine phosphoribosyl transferase 1 (*HPRT1*) as internal controls. Data were analyzed by ANOVA with post-hoc Tukey’s test (**P* < 0.05, *****P* < 0.0001). Error bars represent standard deviation (S.D.); n = 13-15.

**Supplemental experimental procedures**

**Detection of *S*-palmitoylation by acyl-RAC**

The acyl-RAC method was applied essentially as described (3) with minor modifications. Cells in a 6-well plate were harvested and lysed in 400 μl of thiol blocking buffer (100 mM HEPES, 1 mM EDTA, 2.5% SDS, 0.1% *S*-methyl methanethiosulfonate (#64306, Sigma-Aldrich, St. Louis, MI), pH 7.4), disrupted by sonication, and incubated at 50 °C for 10 min. Following two acetone precipitations, the pellets were washed with 70% acetone and resuspended in binding buffer (100 mM HEPES, 1 mM EDTA, 1% SDS, pH 7.4). Total protein was quantified with a bicinchoninic acid assay (BCA; Pierce) using BSA as the standard, and 100 μg protein was rediluted in 150 μl of binding buffer. 20 μg of protein from each sample was retained to assess input. When used, an equal volume (150 μL) of freshly prepared 1 M NH_2_OH, pH 7.4, was added followed by 30 μl of prewashed thiopropyl-Sepharose 6B (GE HealthCare Life Sciences, Chicago, IL). Binding was carried out on a rotator at room temperature for 2 h. The resin was washed four times with binding buffer and eluted in 25 μl of SDS sample buffer containing 1% 2-mercaptoethanol at 50 °C for 10 min prior to SDS-PAGE and Western blotting.

**Detection of *S*-palmitoylation by metabolic labeling and click chemistry**

Metabolic labeling and click chemistry were carried out essentially as described (4) with minor modifications. HEK293 cells expressing HA-mouse zDHHC5 wild-type and mutants in a 6-well plate were incubated with 50 μM 17-octadecynoic acid (34450-18-5, Cayman Chemical, Ann Arbor, MI) in a growth medium for 4 h. Cells were then washed three times with ice-cold PBS and solubilized through sonication in HN buffer (50 mM 4-[2-hydroxyethyl]-1-piperazineethanesulfonic acid, 150 mM sodium chloride, pH 7.4) containing protease inhibitor cocktail (03969-21, Nacalai tesque, Kyoto, Japan). To concentrate the membrane fraction, cell lysates were centrifuged at 20,000 g for 15 min at 4 °C. The pellets were resuspended in HN buffer containing 4% SDS and subjected to methanol‒chloroform precipitation to remove the excess probe and other probe-incorporated metabolites. The pellets were then resuspended in 1% SDS in HN buffer. After measuring the protein concentration using the BCA protein assay, lysate containing 150 μg of membrane protein was adjusted to a reaction volume of 150 μl containing 100 μM azide-PEG3-biotin (762024, Merck, Darmstadt, Germany), 1 μM neutralized tris(2-carboxyethyl)phosphine (77720, Thermo Fisher Scientific), 83.5 μM Tris[(1-benzyl-1H-1,2,3-triazol-4-yl)methyl]amine dissolved in DMSO/tert-butanol (20:80%, 678937, Merck), and 1 μM CuSO_4_ (final reagent volume adjusted with HN buffer) and incubated for 1 h at room temperature. The proteins were then precipitated with chloroform‒methanol, and the resultant pellets were dissolved in 40 μl of 1% SDS in HN buffer. A total of 30 μg of solubilized proteins was retained to assess total input, whereas the remaining 120 μg was incubated with 10 μl of streptavidin-agarose (S1638, Merck) in 400 μl of HN buffer for 2.5 h at room temperature. After washing, the biotinylated proteins were eluted with an SDS-sample buffer at 95 °C for 5 min. For immunoblotting analysis, the eluted proteins were separated using SDS-PAGE and visualized with anti-HA antibody.

**Human mRNA data analysis**

The analyses in this study were conducted using publicly available datasets from GEO (Gene Expression Omnibus), which were previously approved by relevant ethical review boards. No additional ethical approval was required for this study.
