# Supplementary figures and images for: A facile assay for zDHHC palmitoyl transferase activation elucidates effects of mutation and modification
Source: J Lipid Res. 2025 Jan 10;66(2):100743. doi: 10.1016/j.jlr.2025.100743 (PMC11870023; doi:10.1016/j.jlr.2025.100743)

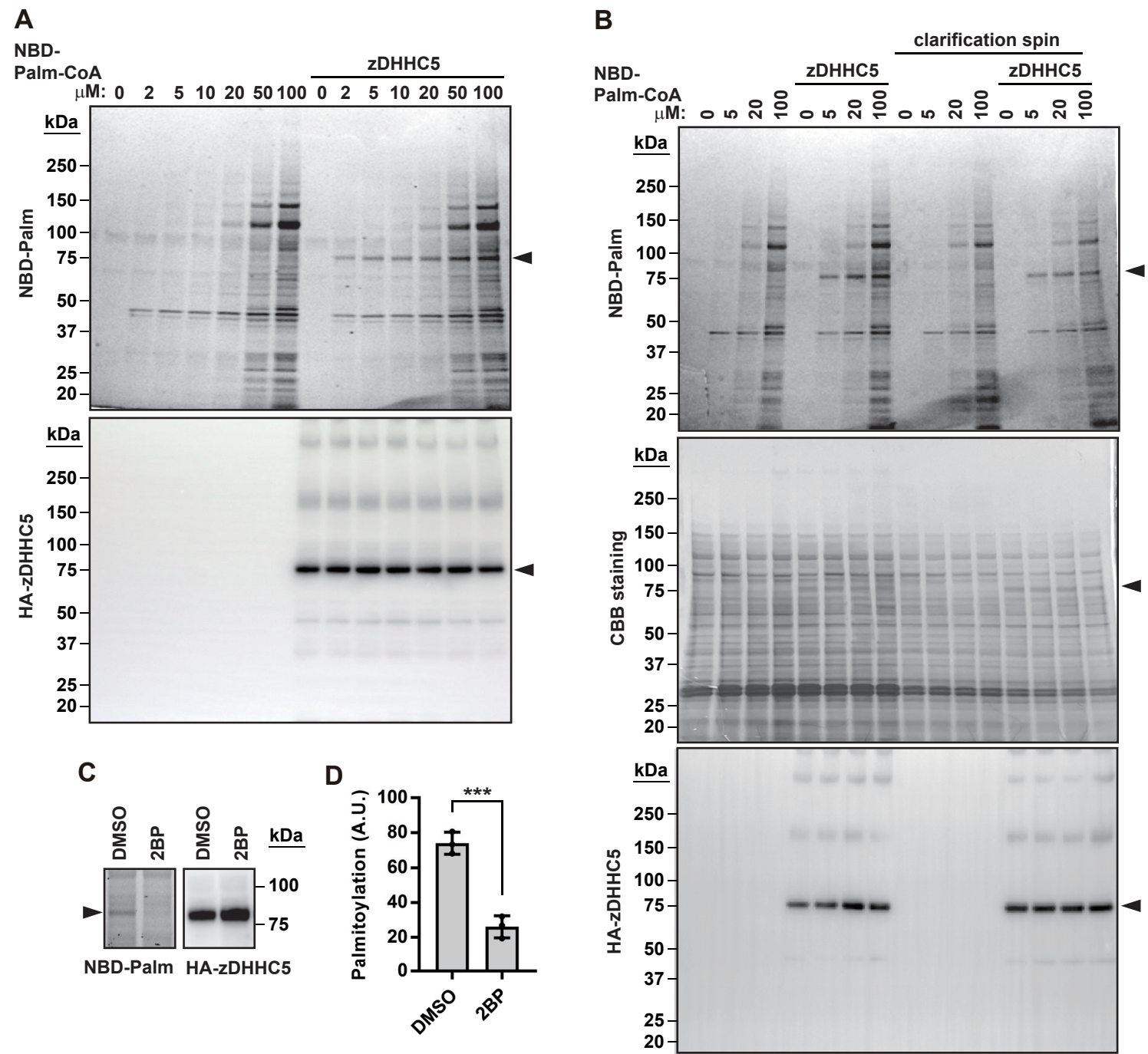

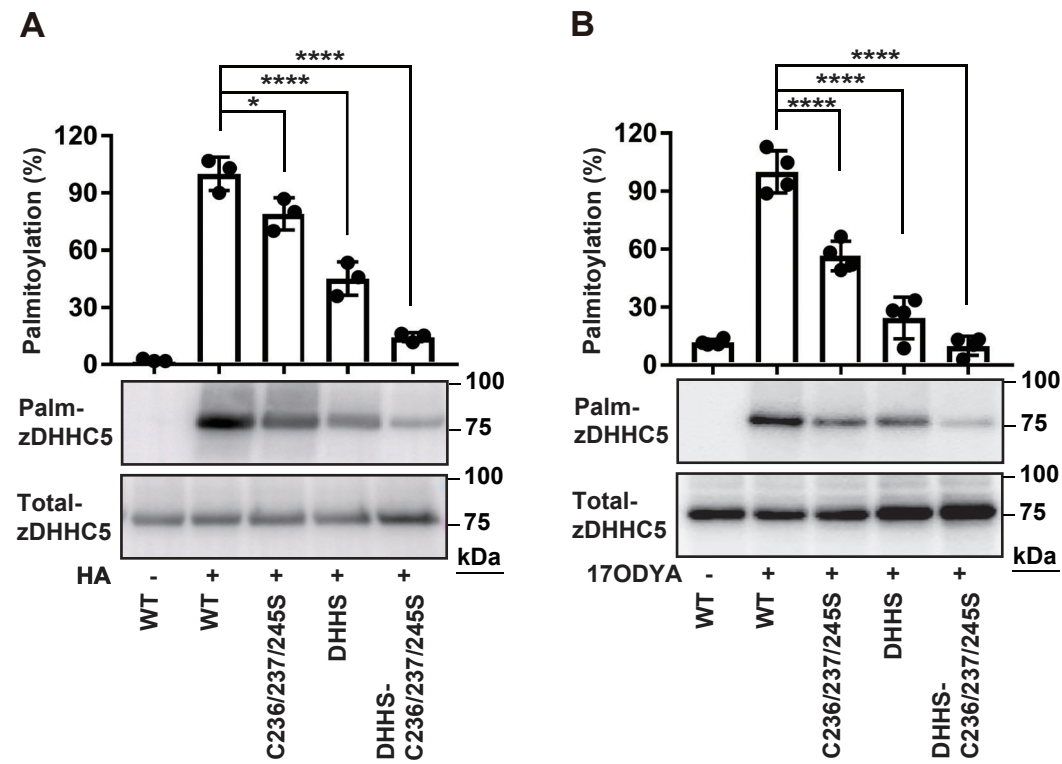

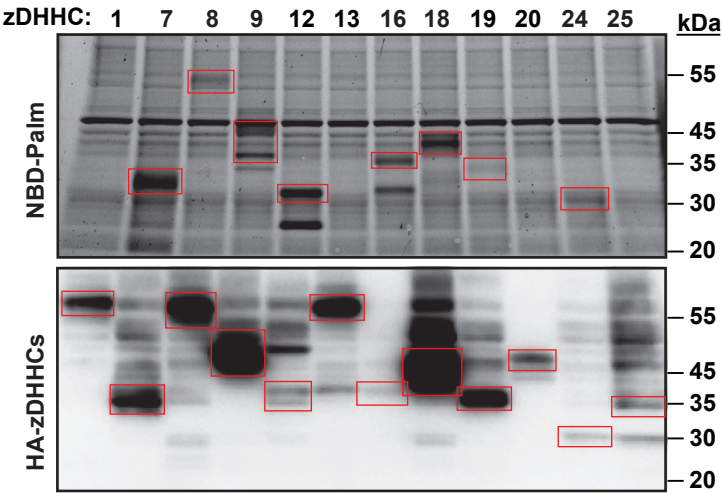

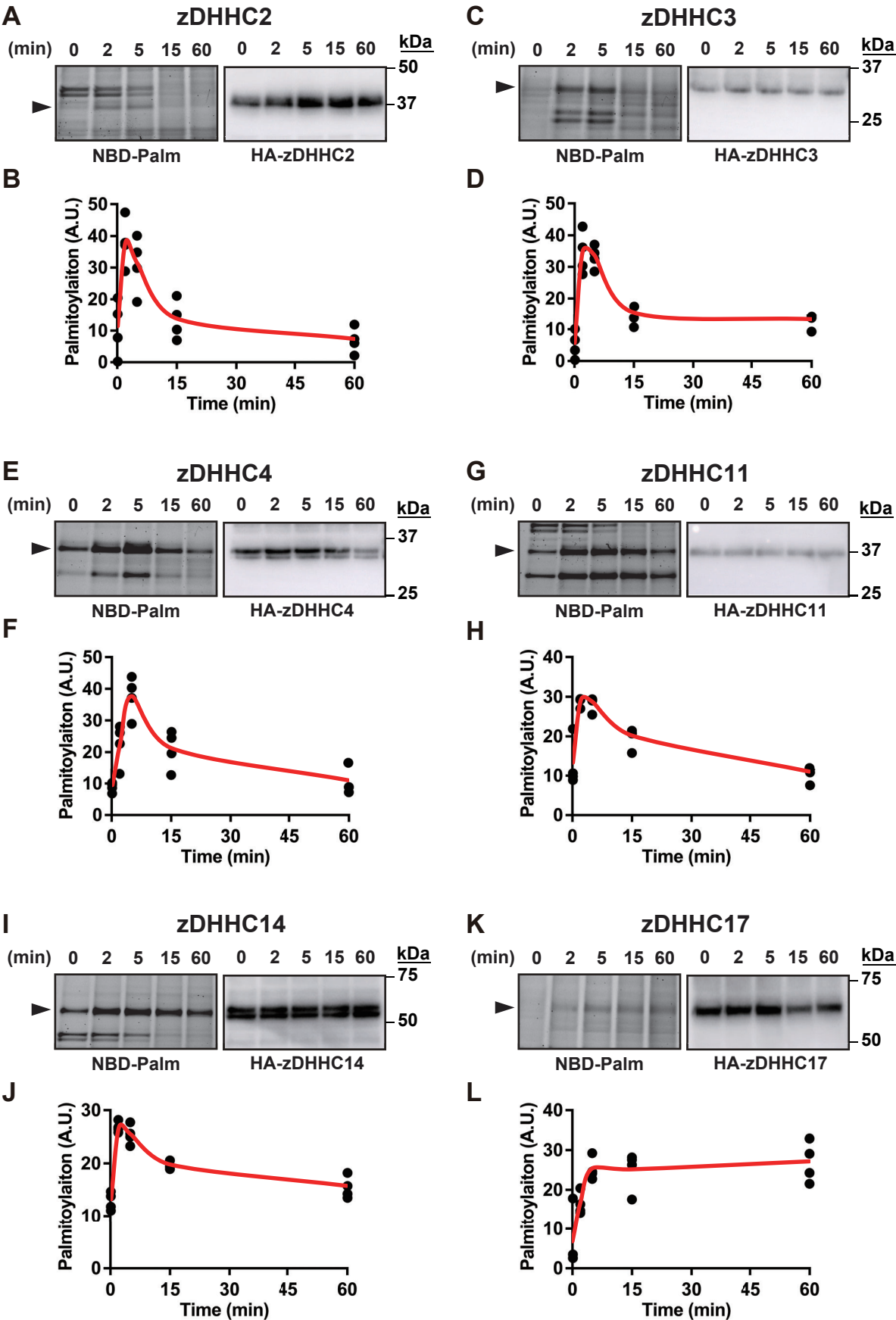

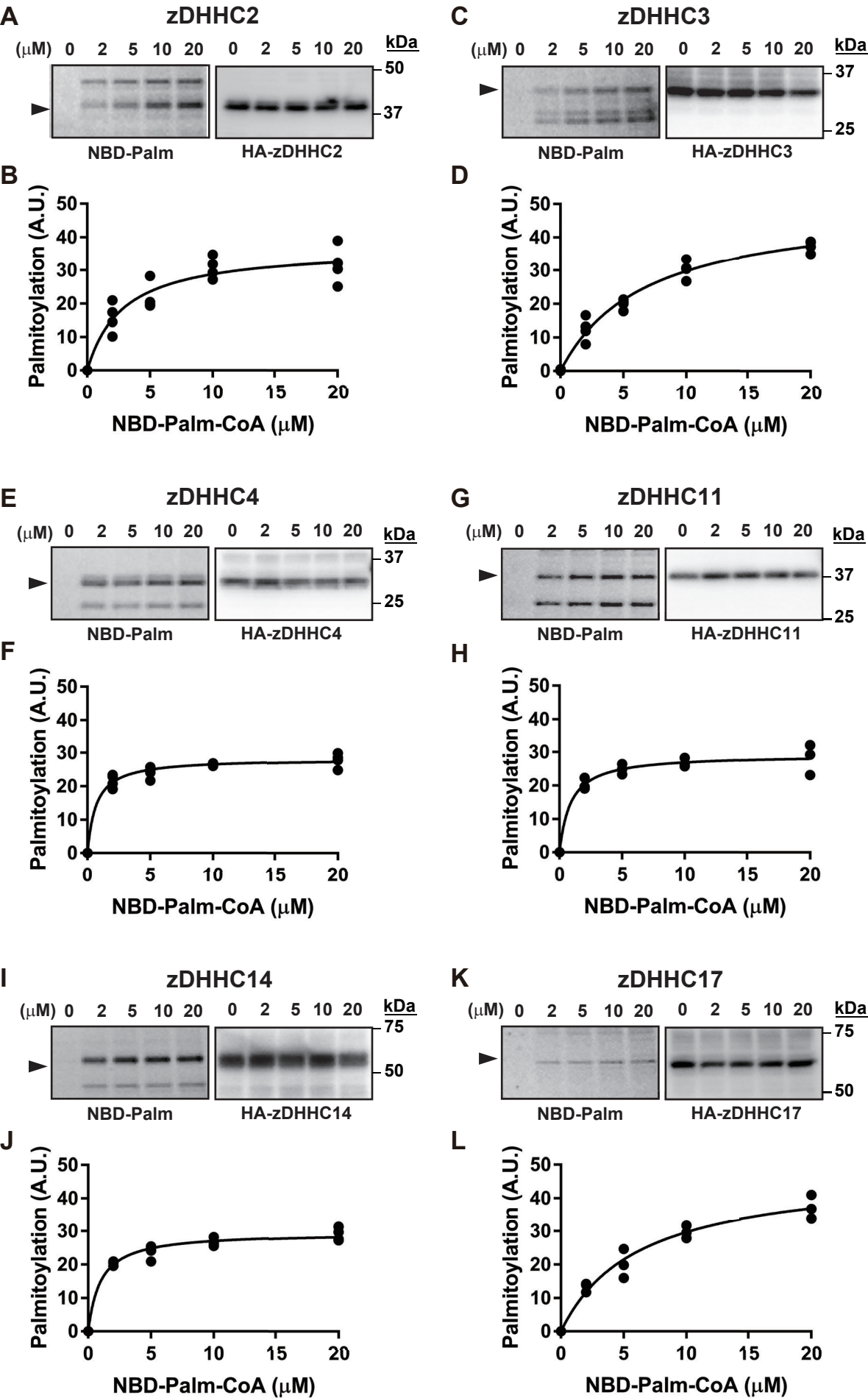

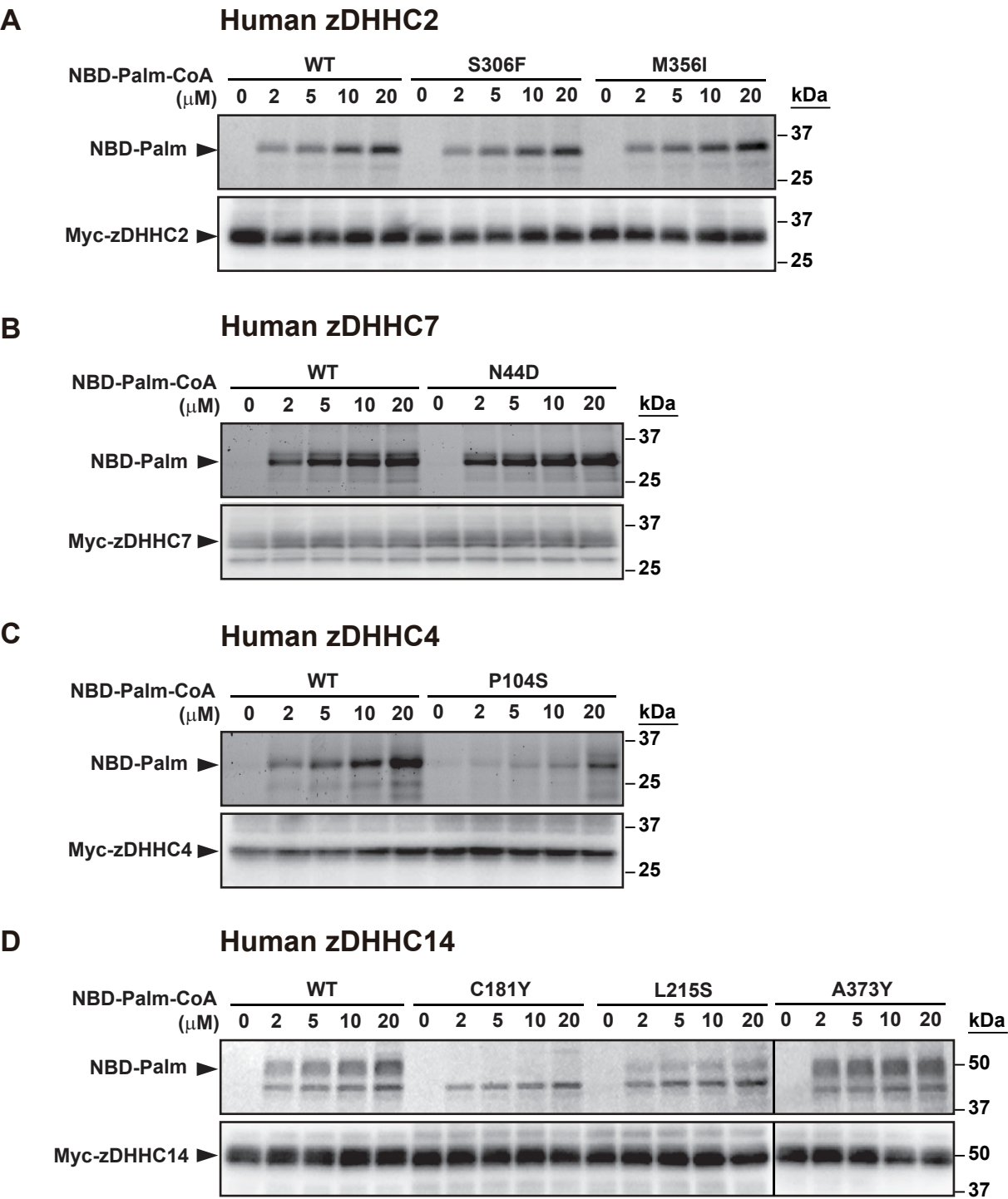

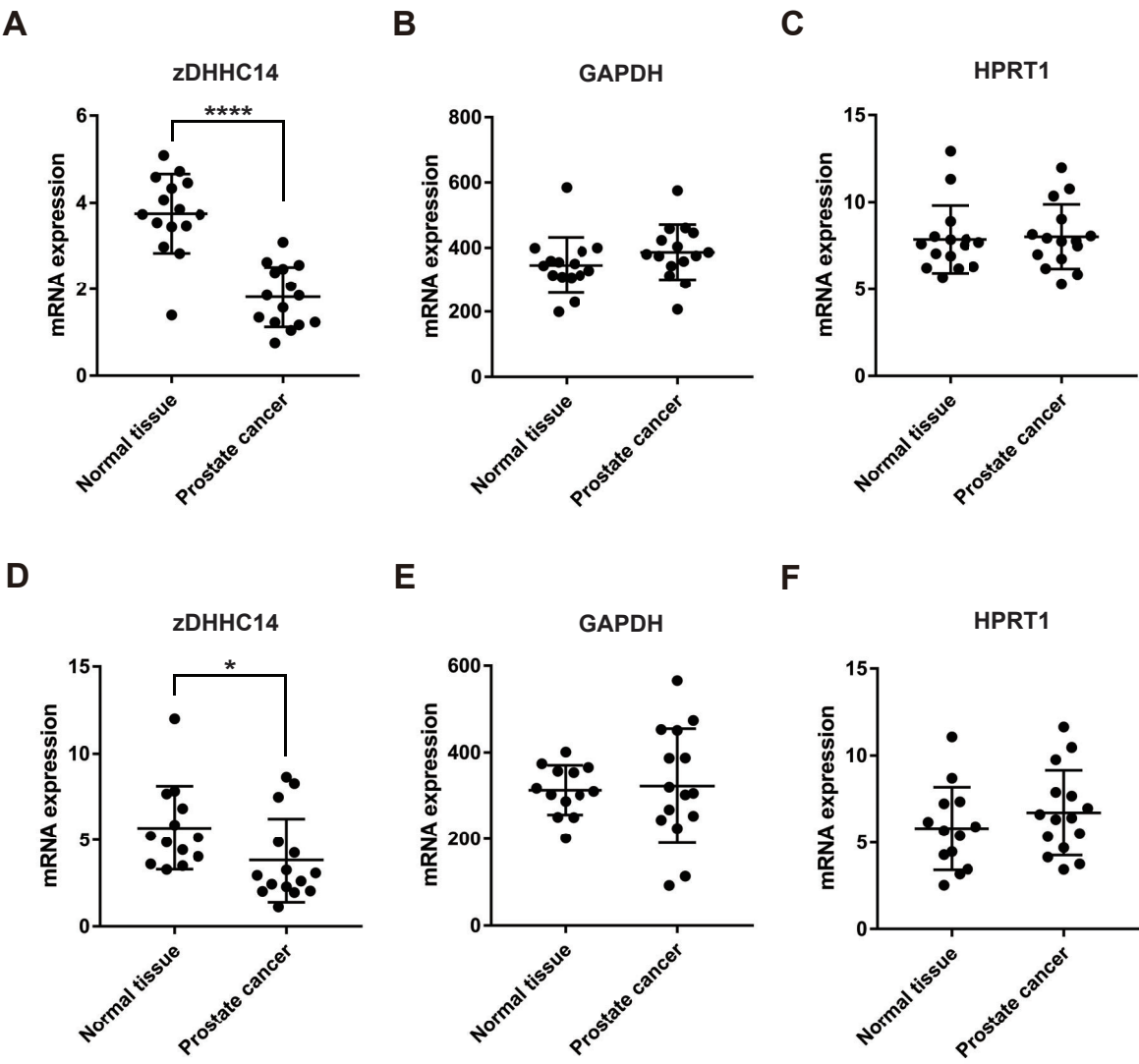

Supplement: Supplemental Figures [file mmc2.pdf]
